# Target-Dependent Expression of IL12 by synNotch Receptor-Engineered NK92 Cells Increases the Antitumor Activities of CAR-T Cells

**DOI:** 10.3389/fonc.2019.01448

**Published:** 2019-12-19

**Authors:** Hong Luo, Xiuqi Wu, Ruixin Sun, Jingwen Su, Yi Wang, Yiwei Dong, Bizhi Shi, Yansha Sun, Hua Jiang, Zonghai Li

**Affiliations:** ^1^State Key Laboratory of Oncogenes and Related Genes, Shanghai Cancer Institute, Renji Hospital, School of Biomedical Engineering, Shanghai Jiao Tong University, Shanghai, China; ^2^State Key Laboratory of Oncogenes and Related Genes, Shanghai Cancer Institute, Renji Hospital, Shanghai Jiao Tong University School of Medicine, Shanghai, China; ^3^CARsgen Therapeutics, Shanghai, China

**Keywords:** IL12, synNotch receptor, NK92, CAR-T, toxicity

## Abstract

IL12 is an immune-stimulatory cytokine for key immune cells including T cells and NK cells. However, systemic administration of IL12 has serious side effects that limit its clinical application in patients. Recently, synthetic Notch (synNotch) receptors have been developed that induce transcriptional activation and deliver therapeutic payloads in response to the reorganization of specific antigens. NK92 cell is a human natural killer (NK) cell line which has been developed as tools for adjuvant immunotherapy of cancer. Here, we explored the possibility of using synNotch receptor-engineered NK92 cells to selectively secrete IL12 at the tumor site and increase the antitumor activities of chimeric antigen receptor (CAR)-modified T cells. Compared with the nuclear factor of activated T-cells (NFATs) responsive promoter, which is another regulatory element, the synNotch receptor was better at controlling the expression of cytokines. NK92 cells transduced with the GPC3-specific synNotch receptor could produce the proinflammatory cytokine IL12 (GPC3-Syn-IL12-NK92) in response to GPC3 antigen expressed in cancer cells. *In vivo* GPC3-Syn-IL12-NK92 cells controlling IL12 production could enhance the antitumor ability of GPC3-redirected CAR T cells and increase the infiltration of T cells without inducing toxicity. Taken together, our results demonstrated that IL12 supplementation by synNotch-engineered NK92 cells could secrete IL12 in a target-dependent manner, and promote the antitumor efficiency of CAR-T cells. Local expression of IL12 by synNotch-engineered NK92 cells might be a safe approach to enhance the clinical outcome of CAR-T cell therapy.

## Introduction

Adoptive cell transfer (ACT)–based immunotherapy with autologous tumor infiltrating lymphocytes has mediated dramatic tumor regressions in patients with melanoma (1, 2). T cells genetically engineered to express chimeric antigen receptors (CAR) constitute the most clinically advanced form of ACT approved to date for the treatment of leukemias and lymphomas (3). At present, two anti-CD19 CAR-T cells have been approved by the US Food and Drug Administration (FDA). CARs are synthetic receptors that are able to confer antigen-binding and activating functions on T cells with the aim of therapeutically targeting cancer cells (4). Despite encouraging success in the hematologic malignancies, however, solid tumors still escape immune detection and elimination because of the immunosuppressive tumor microenvironment. A strategy for overcoming the suppression involves the use of the fourth generation of CARs, which are CAR T cells engineered to constitutively or inducibly express proinflammatory cytokines (5). One such candidate is Interleukin 12(IL12), which strongly enhances the response of innate and adoptive immune cells to cancer cells (6).

IL12, a well-known potent proinflammatory cytokine composed of p35 and p40 subunits, has biological significance in both adaptive and innate immunity (6, 7). IL12 has many functions such as reactivation of tumor infiltrates lymphocytes (TILs), inhibition of Treg-mediated suppression of effector T cells and recruitment of NK cells to the tumor site, which can promote antitumor responses and regulate the tumor environment (8–10). The effectiveness of IL12 in mediating tumor regression either alone or in conjunction with IL2 in various animal models (11, 12) has led to the testing of IL12 in cancer patients in clinical trials beginning in 1994 (13). However, systemic IL12 application induces severe toxicity, including adverse intestinal, hematopoietic, pulmonary, and hepatic effects (14), which results in limitations in clinical use at therapeutically effective doses (14–16). For the sake of limiting systemic toxicity of IL12 (17), T cells were transduced with an expression cassette using a nuclear factor of activated T cells (NFAT)- inducible promoter to produce IL12 at the tumor site locally (18, 19). NFAT is the first discovered transcription factor family that binds to the IL2 promoter in the process of T cell activation. However, significant toxicities were observed in patients receiving human tumor-infiltrating lymphocytes (TILs) cell doses (0.3 × 10e9 or greater) capable of causing tumor regression (20). There was an unpredictability of peak serum levels of IL12 which might be due to the random introduction of the IL12 gene into T cells with other reactivities such as those against viral antigens capable of stimulating the cells *in vivo*. Therefore, how to control the production of IL12 locally in tumor sites is still a major issue that needs to be addressed.

Recently, a novel customized receptor called synthetic Notch (synNotch) has been developed to control the programming of both input and output upon recognition of user-specific antigens by releasing and activating intracellular transcription factors (21, 22). A synNotch receptor contains an important core regulatory domain derived from the cell receptor Notch, a synthetic intracellular transcriptional domain and a synthetic extracellular recognition domain such as single-chain antibodies (23). After the antigen binding-induced transmembrane cleavage, the intracellular transcriptional domain is released and enters the nucleus, and the target gene expression regulated by the upstream promoter was activated (24). The high programmability of the synNotch receptor leads to the feasibility of delivering therapeutic payloads such as cytokines (including IL12) in a tumor antigen-specific manner. Therefore, using synNotch receptors is an efficient way to ensure safe therapeutic T cell responses (21, 22). However, there are two problems that shall affect the application of synNotch receptors to control IL12 expression in CAR-T cells. One is the size of the CAR plus synnotch receptor; IL12 are very large and will significantly affect the transfection efficiency in T cells. The second one is the high variability of CAR T cell expansion in patients, which may affect the production of IL12.

Natural killer (NK) cells have emerged as tools for adjuvant tumor immunotherapy. The high levels of activating ligands in combination with low expression of inhibitory ligands on target cells contributes to NK cell activation and killing target cells via the perforin granzyme pathway or released proinflammatory cytokines such as interferon-γ (25–27). As autologous NK cells are often obtained from patients, the sufficient source of NK cell lines that can expand continuously made them highly feasible for researchers to investigate novel engineering NK cells (28–30) and potential development for standardized off-the-shelf therapeutics. NK92 is a human NK cell line that consists of a stable and homogenous population and it has been safely used as an allogeneic cell therapeutic among cancer patients with clinical responses (31–33). NK92 cells express multiple activated NK-cell receptors such as NKp46, NKp44, and NKp30, while short on inhibitory KIRs, but the low levels of KIR2DL4 are an exception (34, 35). Moreover, the relatively short lifespan of NK92 cells especially renders these cells safer than T lymphocytes. Based on these properties, NK92 cells may be used as a safe and universal carrier for therapeutic payloads such as IL12.

Therefore, in our study, we built synNotch receptors capable of recognizing glypican-3(GPC3) which demonstrated to be highly expressed in hepatocellular carcinoma (HCC) (36). Previously, we reported that GPC3-targeted CAR-T cells developed by our laboratory were well tolerated in the phase I clinical trial for HCC patients (NCT02395250) (37, 38), indicating that GPC3 is a relatively safe target. In this study, NK92 cells engineered with anti-GPC3 synNotch receptors expressing single-chain IL12 were designated as “GPC3-Syn-IL12-NK92.” In those NK92 cells, IL12 expression is under the control of synNotch receptor. We investigated whether IL12 protein could be delivered to ideal tumor sites by adoptive transfer of GPC3-Syn-IL12-NK92 cells and determined the immune-stimulatory properties of IL12 delivered by GPC3-Syn-IL12-NK92 cells to improve the antitumor ability of GPC3-specific CAR-T cells, with the aim of developing a safe and accurate cytokine administration for tumor treatment.

## Materials and Methods

### Cell Lines

Huh7 cell line was obtained from the RIKEN Cell Bank. 293T, NK92 and other HCC cell lines (PLC/PRF/5, and SK-Hep-1) were purchased from the American Type Culture Collection (ATCC). Human NK92 cells were maintained in alpha minimum essential medium (GIBCO, USA) as described previously (39). SK-Hep-1 cells were lentivirally transduced to stably express human GPC3 using Pwpt-GPC3 lentiviral vectors (designated as “SK-Hep-1-GPC3”). 293T and HCC cells were cultivated in DMEM medium (GIBCO, USA) and 10% fetal bovine serum (FBS, GIBCO, USA).

Human peripheral blood mononuclear cells (PBMCs) were derived from healthy donors. Primary human T cells were isolated from PBMCs by density gradient centrifugation. Then T cells were stimulated with tosyl-activated paramagnetic beads that were immobilized with anti-CD3/anti-CD28 antibodies (Invitrogen) for 24 h and the cell: bead ratio was 1:1. T cells were cultured in human T cell medium AIM-V(GIBCO, USA) with 2% human AB serum and 300 IU/mL recombinant human IL2.

### GPC3 CAR/synNotch Construct Design

The construct of GPC3 CAR was generated according to a previous report by our laboratory (37). The GPC3 synNotch receptor was designed by fusing anti-GPC3 scfv to the intracellular domain of the Notch core fused to the Gal4VP64 transcription factor. To easily determine surface expression, all synNotch receptors contain a myc tag. The receptors were then cloned into the lentiviral vector PHRSIN which contains a PGK promoter used for all NK92 cell experiments. The modified vector PHRLSIN was also used for the response element which contains the Gal4 inducible promoter allowing the expression of genes of interest such as BFP or human IL12. To easily identify transduced NK92 cells, all the response element plasmids contain mCherry, whose expression is constitutively driven by a PGK promoter.

### Lentivirus Production and NK92 Cell Transduction

293T cells were transfected using polyethyleneimine (PEI) with the packaging constructs including PAX2 and pMD2.G, and the pHR transgene expression vector. Six hours post-transfection, the medium was replaced with pre-warmed DMEM containing 2% FBS. Lentiviral supernatant was collected 3 days later and filtered through a 0.45-μm syringe (Millipore, USA), and then concentrated with polyethylene glycol. NK92 cells were infected by spinoculating at 1,800 g at 32°C for 90 min at an optimal multiplicity of infection (MOI) of 20–30 lentiviral particles per cell in 24-well plates containing 2–5 × 10^5^ NK92 cells in each well and 8 μg/ml polybrene (Sigma, USA), 6 μM BX795 (Invitrogen, USA) and 300 IU/ml rhIL2. Infected cells were incubated overnight at 37°C and 5% CO_2_; the supernatant containing the virus was then removed, and fresh medium was added.

### *In vitro* Stimulation of Engineered NK92 Cells

For all NK92 cell stimulations *in vitro*, 2 × 10^5^ NK92 cells expressing GPC3-specific synNotch receptors were incubated with various target cells at an effector to target (E: T) ratio of 1:1 in 24-well culture plates. To force the interaction of these co-cultured cells, the plates were centrifuged at 400 g for 1 min, and the cultured cells were harvested and analyzed by flow cytometry after 24 h of co-culture for reporter gene (GFP or mCherry) expression.

### Cytokine Release Assays

SynNotch NK92 cells were stimulated with different cancer cell lines for various time periods, and supernatant was harvested at different time points. The supernatants were centrifuged at 300 g for 10 min, and cytokines secreted by NK92 cells were examined using a commercially available enzyme-linked immunosorbent assay (ELISA) kit (Multi Sciences Biotech, Hangzhou, China).

### *In vitro* Cytotoxicity Assays

To study the cytotoxicity of genetically modified T cells (GPC3-28Z) when co-cultured with GPC3-Syn-IL12 NK92 at a ratio of 1:1, different HCC cells were co-cultured with GPC3-28Z CAR-T cells at an E:T ratios of 3:1, 1:1, and 1:3. After 12 h of co-culture, the specific cytotoxicity of GPC3-28Z CAR-T cells was monitored by the LDH release in the supernatants using the CytoTox 96 Nonradioactive Cytotoxicity Kit (Promega, Madison, WI).

### *In vivo* Tumor Growth Delay Experiments

Experiments on 6- to 8-week-old immunodeficient NOD.Cg-Prkdc^scid^ Il2rg^tm1Wjl^/SzJ (NSG) mice were performed in accordance with the Experiment Animal Care Commission of Shanghai Cancer Institute and housed under specific pathogen-free conditions at the Shanghai Cancer Institute Experimental Animal Center (Shanghai, China). All mice were injected on day 0 with 2 × 10^6^ Huh-7 cells on their right flank for establishing subcutaneous (s.c.) Huh-7 models. After 18 days of tumor growth when the tumor volume reached approximately 100 to 200 mm^3^, mice were divided into four groups (*n* = 6) according to the average tumor volume and injected intravenously (i.v.) with the following CAR-T cells or NK92 cells: (1) untreated T cells (UTD) in sterile PBS; (2) 1 × 10^6^ GPC3-Syn-IL12 NK92 cells in sterile PBS; (3) 1 × 10^6^ GPC3-28Z CAR-T cells in sterile PBS; (4) both 1 × 10^6^ GPC3-28Z CAR-T cells and 1 × 10^6^ GPC3-Syn-IL12 NK92 cells in sterile PBS. Treatment of 1 × 10^6^ GPC3-Syn-IL12 NK92 cells was repeated every 2–3 days. The tumor growth was measured by calipers twice a week, and tumor volumes were calculated on the basis of: volume = length x (width)^2^ × 0.5. All of these mice were euthanized when the mean tumor volume reached 1,500 to 2,000 mm^3^ in the control mice.

### Immunohistochemistry and Histopathological Analysis

Tumor tissues and organs were resected from mice and fixed with formalin and embedded in paraffin and then prepared as 3-mm-thick sections. The organ slides were directly stained with HE. The tumor tissue sections were stained for the presence of human T cells using a mouse monoclonal anti-human CD3ε antibody (Thermo Scientific) and the proliferation of tumor cells using a mouse anti-human Ki67 antibody (Abcam). Following incubation with the primary antibody overnight at 4°C, the secondary antibody was added and the results were visualized using a ChemMate Envision Detection Kit (DakoCytomation).

### Statistics

All experiments were performed at least three times and all data were analyzed using GraphPad Prism 5.0. Data (tumor volume, tumor weight and body weight) are presented as the mean ± SEM. Statistical significance of differences between groups was analyzed by two-tailed Student's *t*-tests, one-way ANOVA and Tukey's test. ^*^p < 0.05, ^**^p < 0.01 and ^***^p < 0.001 were considered statistically significant.

## Results

### Construction and Comparison of GPC3-Specific Synnotch Receptor and NFAT Responsive Promoter in NK92 Cells

**Figure 1:**
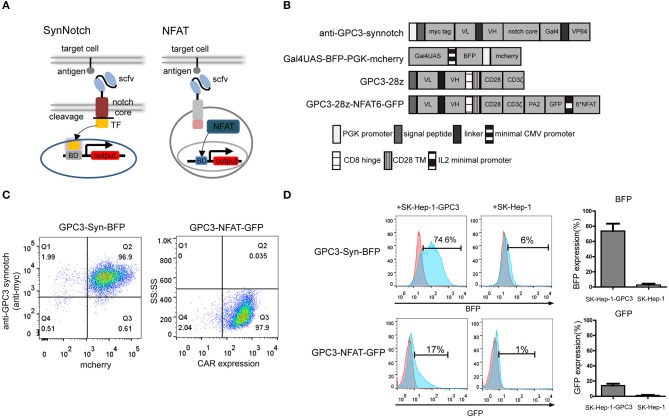
Construction and comparison of GPC3-specific synNotch receptor and NFAT-responsive promoter in NK92 cells. **(A)** Schematic representation of the synNotch and NFAT regulation system and the lentiviral vectors **(B)**. In the anti-GPC3-synNotch vector, the anti-GPC3 scfv is linked to the Notch core and the transcription factor. The reporter Gal4UAS-BFP-PGK-mCherry vector encodes BFP under the Gal4UAS promoter. The GPC3-28Z consisted of a CD8α signal peptide (SP), a humanized GPC3-specific signal chain antibody fragment (scfv), a CD8α signal hinge region and CD28 transmembrane region (TM), followed by the intracellular domains of co-stimulatory CD28 and intracellular domain of CD3ζ. The GPC3-28Z-NFAT-GFP vector expresses GFP under the transcriptional control of the inducible minimal IL2 promoter that contains 6 NFAT-binding motifs. **(C)** GPC3-specific synNotch and NFAT NK92 cell expression were analyzed using flow cytometry. CAR expression on the NK92 cells was detected with goat anti-human biotin-conjugated anti-Fab antibody followed by PE-conjugated streptomycin. The data shown are representative with similar results. **(D)** *In vitro* regulator efficiency of NK92 cells with two different regulator systems following activation with specific target cells. The reporter BFP and GFP fluorescence levels after co-culturing of NK92 cells with target SK-Hep-1-GPC3 or SK-Hep-1 cells for 24 h. The histogram shows the expression rate of reporter genes. Each experiment was repeated at least twice with similar results.

The design of the synNotch and NFAT circuits are outlined in Figure 1A. A cell is engineered to express a synNotch receptor that can recognize specific antigen expression on the tumor. In addition, a reporter construct that contains a responsive promoter is also engineered in the cell, and a gene of interest, such as cytokine, would be expressed after the activation by the synNotch-induced transcription factor (40). Here, we generated a functional synNotch receptor using anti-GPC3 scfv as the extracellular domain to recognize the specific GPC3 antigen, and the Notch core region of the receptor was fused to the engineered transcription factor (Gal4VP64). The reporter construct composes a Gal4UAS responsive promoter that controls a gene of interest, such as blue fluorescent protein (BFP) expression. When GPC3 synNotch receptor expressing cells recognize tumor cells expressing GPC3 antigen, the transcription factor Gal4VP64 is separated from the receptor and thereby translocated into the nucleus regulating the expression of the reporter gene.

In addition to synNotch receptor, it has been demonstrated that an NFAT-responsive promoter which that contains six NFAT-binding motifs followed by the minimal IL2 promoter can induce gene expression in activated T cells (18). The lentiviral expression vectors encoding the GPC3-specific synNotch receptor and NFAT responsive promoter are shown in Figure 1B.

First, we compared the two different regulatory systems in NK92 cells. NK92 cells were engineered to stably express the GPC3 synNotch receptor and the corresponding transcriptional response element controlling the expression of BFP using lentivirus which was designated “GPC3-Syn-BFP-NK92.” In brief, GPC3-Syn-BFP-NK92 cells were a double transduction of lentivirus “anti-GPC3-synnotch” and “Gal4UAS-BFP-PGK-mcherry”. In addition, we engineered NK92 cells expressing anti-GPC3 CAR and NFAT-GFP.PA2 that expressing the GFP under the transcriptional control of the NFAT-responsive minimal IL2 promoter, which were named as “GPC3-NFAT-GFP-NK92.” To increase the efficiency of NK92, we sorted the NK92 cells transduced with lentiviral particles using flow cytometry and the expression of the two regulatory systems in the NK92 cells was almost 100% (Figure 1C).

To compare the regulator efficiency, the GPC3-transfected cells (SK-Hep-1-GPC3) and GPC3-negative cells (SK-Hep-1) were used for specific stimulation. The results indicated that GPC3-Syn-BFP NK92 cells drove BEP reporter expression in 70–80% within 24 h of co-culture with GPC3-transfected SK-Hep-1 cells but did not show BFP with GPC3-negative SK-Hep-1 cells. However, GPC3-NFAT-GFP-NK92 drove only slight GFP reporter expression (10–20%) (Figure 1D). These results indicated that the synNotch system was more effective than the NFAT system in driving reporter gene expression in NK92 cells in the presence of target cells. Only limited expression of BFP (5%-8%) in GPC3-Syn-BFP NK92 cells in the presence of SK-Hep-1 cells indicated slight leakage expression of the target genes in this system.

### Target-Dependent Expression of IL12 by synNotch Receptor

**Figure 2:**
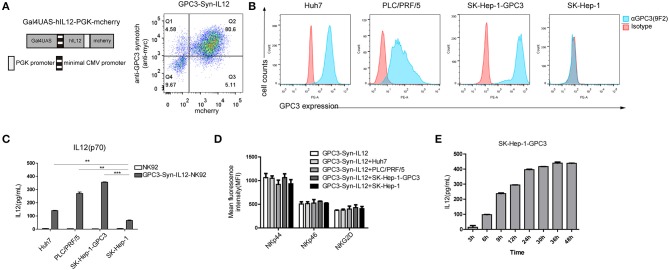
Target-dependent expression of IL12 by the synNotch receptor. **(A)** Schematic representative of GPC3-Syn-IL12 and the transduction efficiency of GPC3-Syn-IL12 in NK92 cells. **(B)** Surface GPC3 expression (blue) on HCC cell lines was detected by FACS analysis. Isotype antibody control (red) served as controls. **(C)** GPC3-Syn-IL12 NK92 cells co-cultured with the indicated target cell lines for 24 h. Culture supernatants were assayed for secreted IL12 by ELISA. **(D)** Flow cytometry analyses of the phenotypic characterization of GPC3-Syn-IL12 NK92 cells co-cultured with different target cell lines for 24 h. Surface expression of NKp44, NKp46 and NKG2D was assessed using mean fluorescence intensity (MFI). The data presented are the quantitative data. **(E)** IL12 levels produced by GPC3-Syn-IL12 NK92 cells stimulated by SK-Hep-1-GPC3 cells after different time points. Each experiment was repeated at least twice with similar results. Data are presented as the mean ± SEM, and significant differences between groups were measured by using one-way analysis of variance (ANOVA). ^**^P < 0.01, ^***^P < 0.001. Only significant differences are indicated.

To control the expression of IL12, we then engineered NK92 cells with anti-GPC3 synNotch receptors to produce IL12 (“GPC3-Syn-IL12-NK92”) in the presence of GPC3-positive tumor cells (Figure 2A). GPC3-Syn-IL12-NK92 cells were a double transduction of lentivirus “anti-GPC3-synnotch” and “Gal4UAS-BFP-PGK-IL12”. Firstly, we compared the IL12 expression between GPC3-Syn-IL12-NK92 cells and GPC3-NFAT-IL12-NK92 cells which were transduction of lentivirus “GPC3-28z-NFAT6-IL12” (Figure S1A). Consistent with previous results, GPC3-Syn-IL12 NK92 cells drove more IL12 secretion but GPC3-NFAT-IL12-NK92 cells did not (Figure S1B). GPC3 expression on used HCC cell lines in our study was analyzed by flow cytometry (Figure 2B) and we also detected the HLA and NKG2D ligand expression on the HCC cell line Huh-7 (Figure S2) in order to prove that GPC3-Syn-NK92 cells may not be activated by these tumor characteristics. As expected, these transduced NK92 cells selectively activated and secreted IL12 in response to the GPC3-positive tumor cells (Figure 2C), while only very limited expression could be observed in the presence of GPC3-negative SK-Hep-1 cells. We next investigated whether the expression of IL12 controlled by anti-GPC3 synNotch receptor could change the NK92 cell phenotype. Flow cytometry analysis showed that there were no differences in the expression of NK cell receptors NKp44, NKp46 and NKG2D in the presence or absence of GPC3-positive tumor cells (Figure 2D). Then, we tested whether GPC3-Syn-IL12 NK92 cells secrete IL12 depending on the stimulation time. We used GPC3-positive tumor cells SK-Hep-1-GPC3 to stimulate NK92 cells and found that GPC3-Syn-IL12-NK92 cells secreted an increasing amount IL12 with the extension of co-culture time and IL-12 expression reached steady-state expression by 24 h (Figure 2E).

### Increased Cytokine Release by CAR-T Cells in the Presence of GPC3-Syn-IL12-NK92 Cells

**Figure 3:**
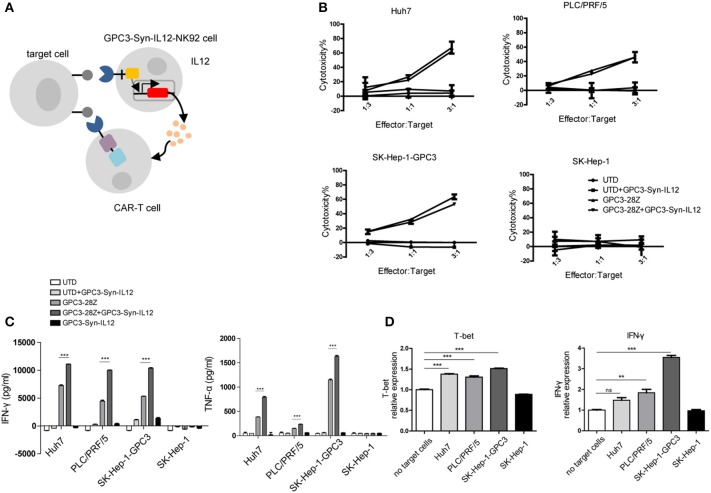
*In vitro* activities of CAR T cells in the presence of GPC3-syn-IL12-NK92 cells. **(A)** NK92 cells were engineered with the α-GPC3 synNotch controlling the expression of IL12 in response to GPC3 antigen on the tumor surface. The IL12 secretion effect on GPC3-28Z CAR T cells may influence antitumor ability. **(B)** The effector cells (GPC3-28Z CAR T) were co-cultured for 12 h with target cells (1 ×10^4^) at E:F ratios of 3:1, 1:1, and 1:3 in a total volume of 100 μl, and GPC3-Syn-IL12 NK92 cells were the same as GPC3-28Z CAR T cells. Cell lysis was determined by an LDH release assay. **(C)** IFN-γ and TNF-α production by GPC3-28z CAR T and GPC3-Syn-IL12 NK92 cells co-cultured with the indicated HCC cell lines at an E:F ratio of 1:1 for 24 h. **(D)** The expression of T-bet and IFN-γ mRNA in GPC3-28Z CAR T cells by qPCR. Using a 3-μm pore size in 12-well transwell chambers, GPC3-Syn-IL12 NK92 cells were stimulated with different HCC cell lines to secrete IL12 in the upper chambers, and GPC3-28Z CAR T cells were plated in the lower chambers. After 24 h, GPC3-28Z CAR T cells were collected, and total RNA extracted from GPC3-28Z CAR T cells was reverse transcribed and amplified using primers specific for T-bet and IFN-γ. Each experiment was repeated at least twice with similar results. Data are presented as the mean ± SEM, and significant differences between groups were measured by using one-way analysis of variance (ANOVA). ^**^P < 0.01, ^***^P < 0.001. Only significant differences are indicated.

With successful selective expression of IL12, we evaluated the potential effect of IL12 delivered by GPC3-Syn-IL12 NK92 cells on CAR T cells. Human T cells transduced with GPC3-specific CAR(“GPC3-28Z”) were co-cultured with GPC3-Syn-IL12-NK92 cells. The schematic diagram of the two-cell combined interaction is outlined in Figure 3A. To determine whether GPC3-Syn-IL12-NK92 cells could affect lysis of GPC3-positive HCC cells by GPC3-28Z CAR T cells *in vitro*, cytotoxicity assays were performed by incubating GPC3-28Z CAR-T cells with each of the four HCC cell lines and keeping the ratio of NK92 cells and T cells at 1:1. The results indicated that IL12 secreted by GPC3-Syn-IL12-NK92 cells could not directly influence the cytotoxicity of CAR-T cells (Figure 3B). However, in the cytokine production assay, we found that GPC3-28Z CAR-T cells combined with GPC3-Syn-IL12-NK92 cells produced greater IFN-γ and TNF-α when co-cultured with GPC3-positive cells while no increase in these cytokines was observed when these cells were co-cultured with SK-Hep-1, a GPC3-negative cell line (Figure 3C). These results suggested that GPC3-Syn-IL12-NK92 cells could secrete IL12 in the presence of GPC3-positive cells, which could increase the cytokine production of CAR T cells. Consistently, we also observed that GPC3-28Z CAR-T cells combined with GPC3-Syn-IL12-NK92 cells expressed a high number of Th1 cytokines, T-bet and IFN-γ, as determined by qPCR (Figure 3D).

### GPC3-Syn-IL12-NK92 Cells Increased the *in vivo* Tumor-Suppression Capacity of GPC3-28Z CAR-T Cells

**Figure 4:**
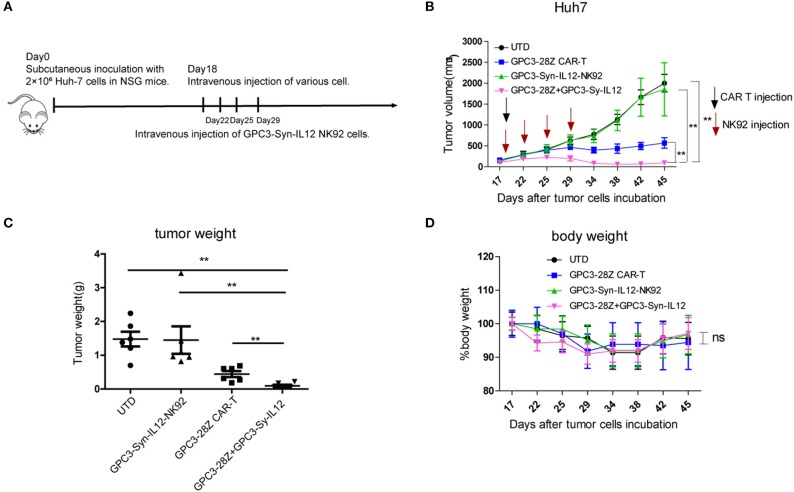
*In vivo* antitumor activities of GPC3-28Z CAR T cells on established subcutaneous GPC3-positive Huh7 xenografts when combined with GPC3-Syn-IL12 NK92 cells. **(A)** Experimental scheme of the *in vivo* antitumor experiment. On day 0, 2 × 10^6^ Huh-7 cells were subcutaneously injected into 6-week-old female NSG mice. On day 18, mice bearing tumors of approximately 150 mm^3^ were infused intravenously with 1 × 10^6^ indicated T cells or NK92 cells. NK92 cells were infused every 2–3 days approximately four times. Each treatment group included 6 mice. **(B)** Tumor growth curve of treatment with the indicated T cells or NK92 cells. At the endpoint, the residual tumors treated with the combination of GPC3-28Z CAR T cells and GPC3-Syn-IL12 NK92 cells were significantly smaller than those in the other control groups. and tumor volumes were calculated on the basis of V = (length×width^2^) ÷ 2. **(C)** Tumor weight at the endpoint of the animal experiment. **(D)** The body weight of each group was measured every 3–4 days (baseline = 100%). Data are presented as the mean ± SEM. Statistical significance was calculated by two-way analysis of variance (ANOVA). ^**^P < 0.01. Only significant differences are indicated.

**Figure 5:**
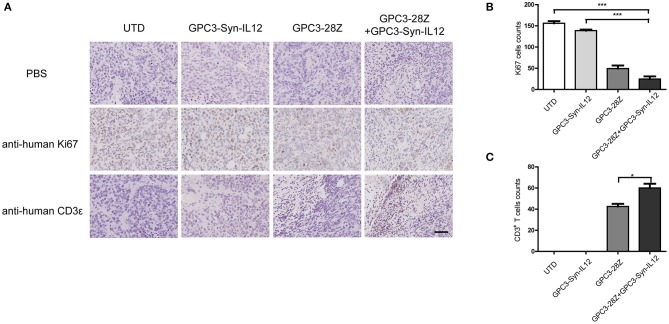
Immunohistochemical (IHC) analysis of Ki67 and CD3 in tumor sections. **(A)** Representative tumor sections stained with Ki67 and CD3 are shown. Tumors were collected from mice bearing subcutaneous xenografts treated with the indicated T cells for 27 days. The images were obtained under 200× magnification. The scale bar was 100 μm. The data shown are representative of experiments with similar results. The images were obtained under 200× magnification. **(B,C)**, Quantification of tumor cells proliferation and human T cells infiltration in tumor sections. The data shown are representative of experiments with similar results. Data are presented as the mean ± SEM. Statistical significance was calculated by two-way analysis of variance (ANOVA). ^*^P < 0.05, ^***^P < 0.001. Only significant differences are indicated.

**Figure 6:**
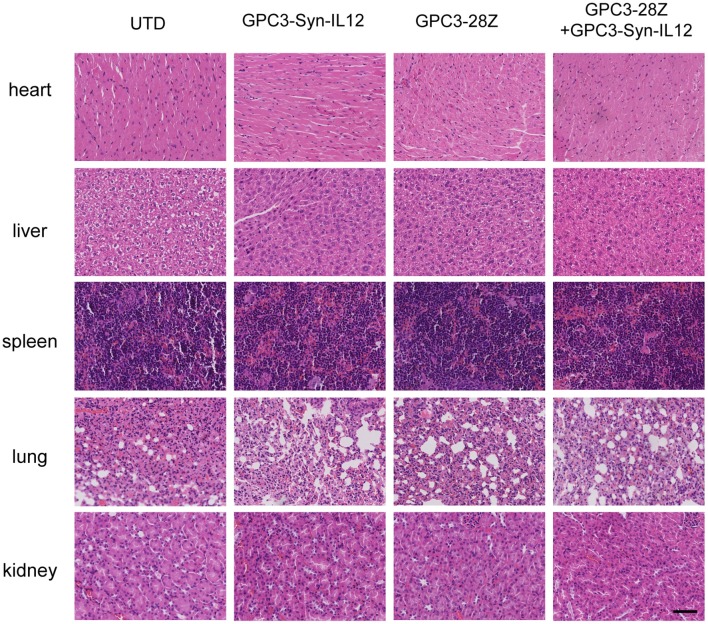
Haematoxylin and eosin (HE) staining of important organs. Organs, including heart, liver, spleen, lung and kidney, were analyzed by HE staining. The specimens were harvested from Huh7-bearing mice sacrificed after study termination. The scale bar was 100 μm. The data shown are representative of experiments with similar results. The images were obtained under 200× magnification.

To further explore whether GPC3-Syn-IL12-NK92 cells could increase the antitumor activities of GPC3-28Z CAR-T cells *in vivo*, a xenograft tumor was established with GPC3^+^ Huh-7 cells in NSG mice. When the tumor volumes reached approximately 200 mm^3^, mice were grouped and treated with GPC3-28Z CAR-T cells or GPC3-Syn-IL12-NK92 cells or their combination in a 1:1 ratio. Synnotch-IL12-NK92 cells were repeatedly administered every 2–3 days for 2 weeks because of the limited lifespan of NK92 cells (Figure 4A). As shown in Figure 4B, mice treated with GPC3-28Z CAR-T cells and their combination with GPC3-Syn-IL12-NK92 cells had an antitumor effect, while no obvious antitumor effect was observed in the mice treated with GPC3-syn-IL12-NK92 alone. The tumors on day 45 in the combination treatment group were significantly smaller than those in the GPC3-28Z CAR-T group (*p* < 0.01) as well as those in the GPC3-Syn-IL12-NK92 group (*p* < 0.001). At the end of the experiments, the tumor weight in the combination group was also significantly less than that of the group treated with GPC3-28Z CAR-T cells (Figure 4C). We also measured the weight of mice in the all therapeutic processes. There were no significant differences in body weights between the different groups (Figure 4D), suggesting IL12 secreted by NK92 cells did not induce systemic toxicity to the mice. At the end of therapy, we harvested xenografts and examined the proliferation of tumor cells and infiltration of T cells. As shown in Figure 5A, a dramatic decrease in the proliferation of tumor cells measured by Ki67 staining and increased infiltration of human CD3^+^ T cells were observed in the combination treatment group, and CD3^+^ T cells that accumulated in the tumor lesions were significantly more than those in the GPC3-28Z CAR-T cell group (Figures 5B,C). Moreover, there was no specific CD3 staining in the tumor sections from mice which treated with UTD and GPC3-Syn-IL12-NK92 cells. In order to explore the kinetics of IL12 expression after CAR-T and NK92 cells infusion, the peripheral blood was collected (Figure S3A). The results shown that the amount of IL12 secretion was related to NK92 cells injection time and gradually reduced with the extension of NK92 cells infusion because of the short lifespan of NK92 cells (Figure S3B). In addition, we elucidated whether IL12 could cause toxicity to the mouse health tissues. We extracted mouse organs such as the heart, liver, spleen, lung and kidney after the treatments for HE staining, and no obvious damage was observed in all groups (Figure 6). These results demonstrated that intravenous administration of GPC3-Syn-IL12-NK92 cells could enhance the antitumor effect of GPC3-28Z CAR-T cells and could result in effective accumulation of CD3^+^ T cells in the tumors without harm to important organs.

## Discussion

Natural killer (NK) cells have emerged and been developed as tools for adjuvant immunotherapy of cancer. Like T cells, NK cells can be modified with chimeric antigen receptors (CARs) that recognize antigens expressed by tumors combined with signaling components that enhance NK cell activity. Multiple studies have shown preclinical success of CAR-expressing NK cells targeting a broad range of tumor antigens NK. Apart from donor-derived primary NK cells, NK92, an established NK cell line, has shown safety and efficiency in allogeneic cell therapy, where clinical responses have been observed in some treated cancer patients (31–33). The results of clinical trials have shown the general safety and efficiency of infused NK-92 cells (31). Here, we engineered NK92 cells with anti-GPC3 synNotch receptors to control the IL12 expression and explored their function on the potential to promote the antitumor activities of anti-GPC3 CAR-T cells.

IL12 plays a significant role in activating antitumor immunity in innate and adaptive immune systems. However, recombinant IL12 treatment is limited by toxic side effects including deaths. For the sake of safety, the systemic administration of IL12 at therapeutic levels was not always allowed in patients with cancer (14–16). Previously clinical trials have explored alternative strategies to deliver IL12 in order to control IL12 delivery to the tumor lesion locally, including transferring IL12 gene-transduced tumor cells (41), fibroblasts (42), or dendritic cells (43). However, the efficacy of these strategies was minimal, highlighting the need to combine IL12 with other antitumor strategies. For example, IL12 together with granulocyte macrophage colony-stimulating factor loaded on microspheres can slow release to the environment (9). Compared with those previously mentioned strategies, target-dependent expression of IL12 by synNotch receptor-engineered NK92 cells has the advantage of delivering IL12 in a controlled fashion. The synNotch receptor, which functions as an additional environmental sensing modulator is a promising specific system to regulate anti-tumor responses in a more efficient and safe way (21).

In this study, we first compared synNotch receptors in driving target gene expression in NK92 cells with NFAT inducible promoters, which are a family of transcription factors identified in activated T cells. The results indicated that the synNotch receptor drove a higher level of reporter gene expression in response to specific antigen GPC3 than the NFAT inducible promoter did. Our further study revealed that GPC3-Syn-IL12 NK92 cells secreted IL12 in a time-dependent manner after exposure to GPC3 antigen-positive tumor cells. But there is some leakiness in response to GPC3-negative tumor cell SK-Hep-1 (Figure 2C). In the previous study, Lim et al. (40) have also observed background activity and discovered that adding the EGF repeat could decrease the basal activation of receptors or changing the amount of receptor expressed could control the output. We needed to improve the synNotch receptor expression in NK92 cells to avoid the basal activation in the future. Though basal activation may induce the expression of IL12, we did not find the influence of leakiness to cytotoxicity and inflammatory cytokines (Figures 2B,C) that may not affect the toxicity profile.

IL12 has been reported to improve cell-mediated immune activity through stimulating cytotoxicity of NK cells and CD8 T lymphocytes (6, 7, 44).We explored whether GPC3-Syn-IL12-NK92 cells secreting IL12 could enhance GPC3-28Z CAR-T cell antitumor activities. The mechanism of CAR T cell therapy is that T cells expressing the CAR can kill cells expressing target antigen directly. In an *in vitro* cytotoxicity assay, GPC3-Syn-IL12-NK92 cells had no evidence of an effect on the cytotoxicity of CAR-T cells, but making CAR T cells produces higher amounts of IFN-γ and TNF-α. We hypothesized that IL12 secreted from NK92 cells inducing multiple inflammatory cytokines producing could overcome tumor cell-mediated immune suppression and provide a favorable environment *in vivo*. Our results here did indicate that the combined treatment with GPC3-28z CAR-T cells and GPC3-Syn-IL12 NK92 cells had better antitumor activities than the single drug treatment in mice bearing GPC3-positive tumor xenografts. More T cell infiltration was also observed in the combination treatment group than in all the other groups, further supporting that GPC3-Syn-IL12 NK92 cells could increase the antitumor immunity of the GPC3-28z CAR-T cells. Although the mechanism underlying this augmentation of antitumor activities is not yet been completely understood, we speculated that IL12 delivered by NK92 cells to the tumor environment could accumulate and enhance T cells to destroy the tumor *in vivo*. Owing to NK92 cells' limited lifespan, they could disappear relatively rapidly from the circulation. Even though we infused multiple rounds of GPC3-Syn-IL12-NK92 cells *in vivo*, we did not observe toxicity of IL12 in mice.

Previous studies have demonstrated that tumor regression induced by IL12 administration in association with IFN-γ which can affect CD4 and CD8 T cell function (45). Consistently, our study here indicated that GPC3-Syn-IL12-NK92 cells could increase the IFN-γ production, which could induce the PD-L1 expression (46). Since PD-L1 is an immunosuppressive phenotype, the increase in PD-L1 after IL12 treatment may be in conflict with the antitumor effect of IL12. In this regard, it is necessary to control the expression of PD-L1 to maintain the improvement of the immune microenvironment after IL12 treatment. Therefore, the synNotch receptor-engineered NK92 cells secreting IL12 might be combined with PD-1/PD-L1 blockade therapy in the future.

In our study, at least two limitations should be considered. First, we use immunocompromized mice (NSG), which lack an effective immune system, to assess the effectiveness of IL12 in situations similar to the clinical immunosuppressive tumor environment. Second, although the combination of GPC3-28Z CAR T and GPC3-Syn-IL12 NK92 cells has shown higher antitumor activity against Huh7 tumors which possess a high level of GPC3 expression, we do not know whether the antitumor effect is dependent on GPC3 expression level.

In conclusion, we have successfully established synNotch receptor-engineered NK92 cells to locally expression of IL12 in the tumor sites, which could enhance the antitumor activities of CAR T cells without inducing obvious toxicity *in vivo*. This type of target-dependent expression of IL12 may be particularly important because IL12 is potent at driving antitumor ability but is toxic for systemic administration. The inability of CAR-T cell therapy to infiltrate and eliminate solid tumors could be greatly improved by using synNotch receptor-engineered NK92 cells which target-dependent expressing cytokine IL12 to help CAR-T cells prime the local disease environment that make it more susceptible to the CAR-T cell therapeutic response. The combination of these two cells is likely to have a significant impact on increasing the effectiveness of immunotherapy, thus, these systems may be considered to be used in clinical studies.

## Data Availability Statement

All datasets generated for this study are included in the article/Supplementary Material.

## Ethics Statement

The animal study was reviewed and approved by Experiment Animal Care Commission of Shanghai Cancer Institute. Written informed consent was obtained from the owners for the participation of their animals in this study.

## Author Contributions

HL and XW took part in the vector construction, *in vitro*, and *in vivo* experiments. RS and JS took part in *in vivo* analysis. YW took part in the cell culture. YD took part in the lentivirus production. BS helped a lot in the vector construction. YS took part in the flow cytometry. HJ helped a lot in the human T cell isolation and stimulation. ZL is the principle investigator of our research group.

### Conflict of Interest

ZL was employed by company CARsgen Therapeutics and has ownership interests of target-dependent expression of IL12 by synNotch receptor-engineered NK92 cells. The remaining authors declare that the research was conducted in the absence of any commercial or financial relationships that could be construed as a potential conflict of interest.
