# Supplementary material for: Target-Dependent Expression of IL12 by synNotch Receptor-Engineered NK92 Cells Increases the Antitumor Activities of CAR-T Cells
Source: Front Oncol. 2019 Dec 19;9:1448. doi: 10.3389/fonc.2019.01448 (PMC6930917; doi:10.3389/fonc.2019.01448)
Supplement: Supplementary file 1 [file Data_Sheet_1.pdf]

Supplementary. S1

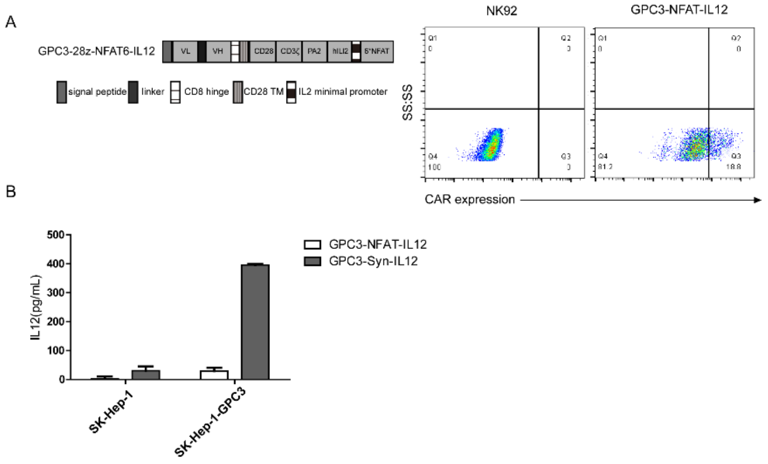

Fig. S1 Construction of GPC3-28z-NFAT6-IL12 and comparison of IL12 expression between GPC3-NFAT-IL12 and GPC3-Syn-IL12 NK92 cells

A, Schematic representative of GPC3-28z-NFAT6-IL12 and the transduction efficiency of GPC3-28z-NFAT6-IL12 in NK92 cells. B, The level of IL12 secretion by GPC3-NFAT-IL12 and GPC3-Syn-IL12 NK92 cells after co-culturing with target cells SK-Hep-1-GPC3 or SK-Hep-1 for 24 hours.

Supplementary. S2

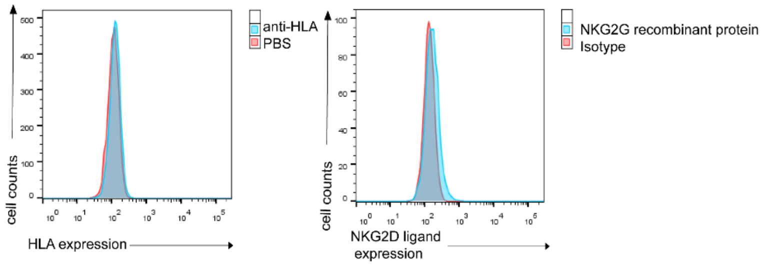

Fig. S2 Surface HLA and NKG2D ligand expression on Huh-7 cell line

Surface HLA and NKG2D ligand expression (blue) on Huh-7 cell were detected by FACS analysis. Huh-7 cell line has no HLA and NKG2D ligand expression on cell surface.

Supplementary. S3

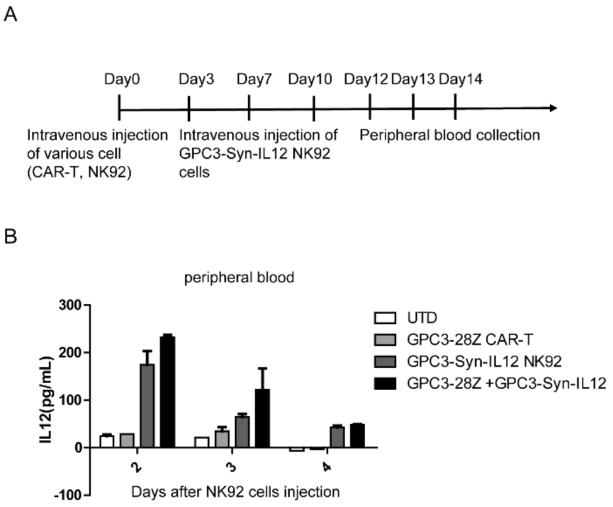

Fig. S3 *In vivo* detection of IL12 secretion

A, The timeline of injection of CAR-T and NK92 cells and collection of peripheral blood. B, The murine serum from peripheral blood on day 2, 3, 4 after the last injection of NK92 cells were collected and the IL12 secretion was detected by ELISA.
